# Nuclear shuttling of CDC4 mediated broad-spectrum antiviral activity against diverse coronaviruses

**DOI:** 10.1080/22221751.2025.2493922

**Published:** 2025-04-22

**Authors:** Mingwei Li, Yang Wu, Jin Tian, Qian Yang, Mingze Gao, Yongrui Wang, Xuepeng Wang, Ju Zhang, Yudi Pan, Hongyan Shi, Da Shi, Xin Zhang, Jianfei Chen, Longjun Guo, Li Feng

**Affiliations:** a State Key Laboratory for Animal Disease Control and Prevention, Harbin Veterinary Research Institute, Chinese Academy of Agricultural Sciences, Harbin, People’s Republic of China; b Laboratory of Medical Genetics, School of Basic Medical Sciences, Harbin Medical University, Harbin, People’s Republic of China

**Keywords:** Coronavirus, CDC4, nuclear shuttling, broad-spectrum, antiviral activity

## Abstract

Pandemics of coronavirus (CoV)-related infection have been a major issue since the outbreaks of SARS, MERS and COVID-2019 in the past decades, leading a substantial threat to public health. Porcine deltacoronavirus (PDCoV), a new swine coronavirus, causes enteropathogenic disease characterized by acute diarrhoea, vomiting and dehydration in suckling piglets and poses potential risks of cross-species transmission. Here we reveal a novel function of CDC4 protein in restricting PDCoV infection. Ectopic expression of CDC4 suppresses PDCoV replication, whereas knockdown of CDC4 expression enhances PDCoV infection. Importantly, it was revealed that PDCoV encoded nucleocapsid (N) was involved in CDC4 nuclear-cytoplasmic shuttling, which was critical for CDC4 to exert the antiviral activity against PDCoV replication. Mechanistically, PDCoV N protein was detected to specifically interact with RIG-I to antagonize RIG-I-like receptor (RLR)-mediated IFN-β production, leading to disruptions of host innate immune defense. Meanwhile, CDC4 was proved to interact with PDCoV N protein and disrupted the interaction between PDCoV N and RIG-I, resulting in alleviated antagonism of IFN-β production mediated by PDCoV N. Similarly, a broad-spectrum inhibitory effects of CDC4 on N mediated antagonism were confirmed by the shared mechanisms among the different coronaviruses from *Coronaviridae* family, such as transmissible gastroenteritis virus (TGEV) from *Alphacoronavirus* (*α*-CoV) and severe acute respiratory syndrome coronavirus 2 (SARS-CoV-2) from *Betacoronavirus* (*β*-CoV). Therefore, a novel antiviral role of CDC4 was elucidated that CDC4 competes binding with CoVs N proteins to suppress CoVs N mediated antagonism of RLR associated signalling pathway in the context of diverse coronavirus infections.

## Introduction

Coronaviruses (CoVs) are a large group of enveloped positive single-strand RNA viruses classified in the Coronaviridae family of Nidovirales order, posing significant public health and economic concerns globally [1]. According to serological and genotypic characterizations, CoVs are divided into four genera, including Alphacoronavirus (α-CoV), Betacoronavirus (β-CoV), Gammacoronavirus (γ-CoV) and Deltacoronavirus (δ-CoV) [1,2]. As the newly emerging swine enteric coronavirus grouped in δ-CoV genus, porcine deltacoronavirus (PDCoV) was first reported in Hong Kong of China in 2012, and additional PDCoV strains have been identified in the United States, Canada, China and South Korea [3–6]. The first PDCoV strain was isolated from intestinal contents of a nursing pig with diarrheal disease in the United States in 2014, and further confirmed enteropathogenic in young pigs, as characterized by severe watery diarrhoea and/or vomiting and severe atrophic enteritis in PDCoV-inoculated pigs [6]. Same as the previous data, PDCoV infection causes a diverse range of clinical conditions including watery diarrhoea, vomiting and dehydration in nursing piglets following challenges with PDCoV NH strain, which was the first Chinese strain isolated in our laboratory in 2016 [7]. Moreover, PDCoV has the possibility of multi-system infection [8,9] and the potential risks of cross-species transmission have been raised by the recent studies, posing a significant challenge to global public health [10].

PDCoV is an enveloped, positive-sense, single-stranded RNA virus. The genome of PDCoV encodes two polyproteins (pp1a and pp1ab) and four major structural proteins (Spike (S), Membrane (M), Envelope (E) and Nucleocapsid (N)) [11]. The innate immune system is established as the first line of host defense to restrict the invading pathogens. It was initiated through the sensing of pattern recognition receptors (PRRs) to the pathogen-associated molecular patterns (PAMPs) and subsequently triggered a series of intracellular signalling. Upon the virus replication, the typical PAMP of viral RNA, is sensed by Toll-like receptor 3 (TLR3) and/or retinoic acid-inducible gene-I (RIG-I)-like receptors (RLRs) (e.g. RIG-I and MDA5) and then recruited to the mitochondrial antiviral signalling protein (MAVS) located in mitochondrial membrane to activate the downstream signalling pathway [12,13]. These signalling cascades are further activated to phosphorylate multiple adaptors (e.g. IRF3, IRF7 and IκBα) by subsequent interactions with TANK-binding kinase 1 (TBK1) and the inhibitor of nuclear factor kappa B (IκB) kinase (IKK) complex, respectively, establishing the strong antiviral state by the production of interferons (IFNs), pro-inflammatory cytokines and a broad range of IFN-stimulated genes (ISGs) [14]. Previous study has proved that the strong response was elicited in PDCoV-infected intestinal enteroids, accompanying the increased levels of IFNs induction, ISGs transcriptions and adaptive immune response post PDCoV infection [15]. After binding their cognate receptors, IFNs activate the JAK-STAT signalling pathway to induce the transcription of diverse ISGs, which play important roles in inhibition of viral infection by targeting multiple stages of the viral life cycle. It has been documented that the mRNA-decapping enzyme 1a (DCP1A) was identified as an ISG and participated in removing the 5'-methylguanosine cap from eukaryotic mRNA to induce the subsequent degradation of mRNA. An apparent increase in DCP1A transcription was confirmed in the context of PDCoV infection and it was involved to decrease the infection of PDCoV [16]. In addition, IFN-induced protein with tetratricopeptide repeats 3 (IFITM3) was induced in IPI-2I cells followed by PDCoV infection and reduced the virus attachment to the target cells by interacting with the S1 subunit of the S protein [17]. Besides the evidence mentioned above, the hosts developed a couple of other strategies to fight against PDCoV infection. PDCoV infection induced the elevated transcriptional levels of phosphoglycerate mutase family member 5 (PGAM5), which function as a serine (Ser)/threonine (Thr) phosphatase in the inner membrane of mitochondrial. It was revealed that PGAM5 was able to activate the IFN signal pathway by interacting with MyD88 and TRAF3, leading to the suppression of PDCoV replication [18]. Based on the increasing evidence, it was demonstrated that the histone deacetylase 6 (HDAC6) was widely involved in the regulation of viral replication [19]. Li et.al demonstrated that HDAC6 plays a negative role in regulation of PDCoV replication by targeted degradation of nsp8 in a deacetylation and ubiquitination dependent manner [19]. Besides the previous reports, further research should be investigated about how the host cells initiate the antiviral signalling to fight against the infection of coronavirus.

CDC4, a component of SCF ubiquitin ligase complex plays important roles in cell growth, survival, differentiation and tumorigenesis via the ubiquitin-proteasome-mediated regulation of protein stability [20]. Two major domains have been well identified in CDC4 sequence: the WD40 domain, which is responsible for substrate recognition; and the F-box domain, which recruits SCF complexes through Skp1 [21]. CDC4 functions as a recognition subunit responsible for ubiquitinating substrate proteins and subsequent targeting them for degradation of multiple proteins including cyclin E, c-Myc, c-Jun, Notch, MCL1, P100 and KLF5 [22-27]. In addition, the antiviral activity of CDC4 was identified during virus infection. It was reported that CDC4 suppresses hepatitis C virus (HCV) replication by binding and degrading the HCV-encoded nonstructural protein 5B (NS5B) in a proteasome-dependent manner [28]. The expression of CDC4 was increased by promyelocytic leukemia protein nuclear bodies (PML-NBs) treatments to exert an antiviral effects against influenza A viruses (IAV) [29]. However, it still remains enigmatic about the questions that whether the antiviral effects of CDC4 was observed in the context of PDCoV infection, and if so, whether there is a novel mechanism to exert the antiviral function of CDC4.

In this study, CDC4 was confirmed to play a negative role in PDCoV replication and a novel antiviral mechanism of CDC4 was dissected. Overexpression of CDC4 suppresses PDCoV replication, whereas knockdown of CDC4 expression increases PDCoV infection. Upon PDCoV infection, the nuclear-to-cytoplasmic shuttling of CDC4 was observed and found to be essential for its antiviral activity. Unexpectedly, The PDCoV N protein facilitates the cytoplasmic translocation of CDC4 in target cells. PDCoV N protein was detected to specifically interact with RIG-I to antagonize RLR-mediated IFN-β production, leading to inhibitions of host innate immune defense. Furthermore, CDC4 disrupted the interaction between PDCoV N and RIG-I, thereby impairing the ability of PDCoV N to antagonize IFN-β production. Similarly, a broad-spectrum antiviral activity of CDC4 was confirmed by using the shared mechanisms among the members in different coronavirus genera from Coronaviridae family, including transmissible gastroenteritis virus (TGEV) from α-CoV and SARS-CoV-2 from β-CoV. Collectively, a novel antiviral role of CDC4 was elucidated that CDC4 competes binding with CoVs N proteins to suppress CoVs N mediated antagonism of RIG-I-like receptor (RLR) associated signalling pathway in the context of diverse coronavirus infections (PDCoV, TGEV, SARS-CoV-2).

## Materials and methods

### Cells and viruses

IPI-2I cells (Porcine intestinal epithelial cells derived from pig ileum), HEK 293 T cells (Human embryonic kidney 293 T cells) and ST cells (Swine testis cells) were cultured in Dulbecco’s minimum essential medium (DMEM) (Gibco, USA) supplemented with 10% heat-inactivated fetal bovine serum (FBS) (Gibco, USA) at 37 °C under 5% CO2. PDCoV strain NH (GenBank accession No: KU981062.1) and TGEV strain AHHF (GenBank accession No: KX499468) were isolated and stored in our laboratory. The Sendai virus (SEV) stock was produced by inoculating the virus into embryonated SPF chicken eggs via the allantoic cavity and collecting the infectious allantoic fluid at 72 h post inoculation. The titre of SEV stock was estimated by hemagglutination (HA) assay using peripheral blood red cells (PBRCs) from SPF chickens. The reciprocal of the highest dilution of the virus capable of causing complete HA was expressed as 1 hemagglutination units (HAU). The titre of SEV stock was calculated as 2^8^ HAU/0.1 mL and was aliquoted to store at −80°C.

### Plasmids

The full length of porcine CDC4 was amplified from IPI-2I cells, and cloned into pCAGGS-HA vector to generate recombinant plasmids (HA-CDC4). Several CDC4 mutants, including the deletion of F-box (HA-ΔF-box) or WD40 (HA-ΔWD40) domain, NES mutation (HA-ΔNES) of four key amino acids (I281G, L284G, L288G, L290G) were designed and cloned into pCAGGs-HA vector using the primers in Table S1. The luciferase reporter plasmids (IFN-β–Luc and pRL-TK), plasmids of critical adaptors in RLRs signalling pathway (Flag/HA-tagged RIG-I, MVAS, TBK1 and IRF3), N plasmids of different CoVs (Myc/Flag-tagged PDCoV-N, Myc-tagged TGEV-N and Myc-tagged SARS-CoV-2-N) were constructed and store in our laboratory. Pierce™ protein A/G magnetic beads were purchased from Thermo Scientific. Lipofectamine RNAiMAX reagent and CDC4 antibody were purchased from Invitrogen. Antibodies against different tags (HA, Myc and Flag), β-actin and GAPDH were purchased from Sigma. Alexa Flour 488 goat anti-rabbit IgG(H + L) or Alexa Flour 594 goat anti-mouse IgG(H + L) was purchased from Invitrogen. IRDye-conjugated secondary antibodies were purchased from Li-Cor Biosciences. The mouse specific monoclonal antibody against PDCoV N was produced and stored in our laboratory.

### Cell transfection and drug treatment

A direct transfection or a bicistronic lentivirus vector pLVX-IRES-ZsGreen1 expression system (Clontech) was introduced to overexpress the target proteins. Cells were either transfected with indicated plasmids using X-tremeGENE transfection reagent according to manufacturer’s instruction (Roche, USA), or transduced with recombinant lentivirus as reported previously [30], followed by subsequent assays at the indicated time points. For some experiments, treatment with nuclear export inhibitor of LMB (2.5 nM) or carrier control (DMSO) were performed in IPI-2I cells as detailed in the corresponding Fig Legends.

### Western blotting assay

The cells were harvested by adding lysis buffer (HaiGene, China) supplemented with a protease and phosphatase inhibitor cocktail (Thermo Scientific, USA). The lysates were separated by SDS-PAGE and the gel transferred to nitrocellulose filter membrane (NC) membranes (Merck Millipore, USA). After blocking, the membranes were then analysed for the expression proteins by using primary antibodies overnight at 4 °C and incubated with an appropriate IRDye-conjugated secondary antibody for 45 min at room temperature.

### Co-immunoprecipitation (Co-IP)

For the Co-IP experiment, the treated cells were harvested before washed with pre-cooled phosphate-buffered saline (PBS; pH 7.4) for 3 times, and lysed with Pierce IP lysis buffer (Thermo Scientific, USA) supplemented with a protease and phosphatase inhibitor cocktail at 4 °C for 30 min. The supernatant was carefully transferred into a new EP tube after centrifugation, adding indicated antibodies according to the antibody instructions overnight at 4 °C, and then incubated with protein A/G beads at 4 °C for 4 h. The immunoprecipitants were washed with IP wash buffer for 5 times, and then subjected to western blotting analysis.

### Immunofluorescence assay (IFA)

The treated IPI-2I cells were fixed with 4% paraformaldehyde for 30 min at 4 °C, and then permeated with 0.2% Triton X-100 for 20 min at room temperature. Cells were stained with mouse anti-PDCoV N monoclonal antibody (MAb) for 1 h at 37 °C, followed by incubation with an appropriate (FITC)-conjugated secondary antibody for another 1 h at room temperature. After washed three times with PBS, the cells were stained with 4’,6-diamidino-2-phenylindole (DAPI) for 5 min at 37 °C. Then, the fluorescence was imaged by laser scanning confocal microscope (Zeiss, Germany).

### RNA interference

For RNA interference, three siRNAs (siCDC4-1295#, siCDC4-1157# and siCDC4-1096#) specifically targeting the porcine CDC4 mRNA were designed and synthesized by Shanghai GenePharma Co.,Ltd (Table S2). IPI-2I cells were transfected with the three siRNAs by using Lipofectamine RNAiMAX reagent according to the manufacturer’s instructions.

### Confocal immunoﬂuorescence microscopy

Following various treatments, the target cell monolayers were washed three times with PBS and ﬁxed in 4% paraformaldehyde for 30 min at 4 °C. Cells were incubated with indicated primary antibodies. After washing, cells were probed with ﬂuorescein isothiocyanate (FITC)-conjugated goat anti-rabbit or anti-mouse IgG (Invitrogen). After stained with DAPI, the cells were examined with a laser scanning confocal microscope (Zeiss, Germany).

### Dual luciferase reporter assay

HEK 293 T cells were seeded in 24-well plates and co-transfected with IFN-β–Luc (0.15 μg), pRL-TK (0.015 μg) together with other indicated plasmids for 24 h. In some stated cases, the cells were inoculated with 40 ul SEV stock for 12 h before the samples were collected for luciferase detections. According to the manufacturer's instructions, luciferase activities were measured with a Dual-Luciferase Reporter Assay System (Promega). The data were shown as relative firefly luciferase activities normalized to renilla luciferase activities.

### Quantitative real-time PCR (RT-qPCR)

Total RNA was extracted from cultured cells using Simply P total RNA extraction kit (BioFlux, China). The cDNA was generated from 2 μg RNA by using Golden MLV Reverse Transcriptase (HaiGene, China). RT-qPCR experiments were performed in three times and normalized to the expression level of β-actin. All the gene-speciﬁc primers are listed in Table S3.

### TCID_50_ assay

Virus samples were frozen and thawed three times and clarified by centrifugation at 8,000×g for 10 min prior to titration. TCID_50_ assay was performed according to the method of Reed & Muench. Briefly, cell monolayers in 96-well tissue culture plates were inoculated with 100 μL each virus stock serially diluted 10-fold for 4 days prior to observation of the presence of cytopathic effect.

### Animal experiment

Six specific-pathogen-free (SPF) pigs were purchased from the Experimental Animal Breeding Center of Harbin Veterinary Research Institute and were randomly assigned into 3 experimental groups (2 pigs per group). For each group, one pig was challenged orally with PDCoV ( 10^7^ TCID_50_) and the other was challenged with DMEM as the mock control. All the pigs were euthanized after the clinical signs of vomiting and diarrhoea were observed at 48 h post infection. Respective tissue samples were collected to detect PDCoV antigens and RNA abundance, and evaluate the expression of endogenous CDC4. The animal experiment was approved by the Harbin Veterinary Research Institute and performed on the basis of animal ethical guidelines and approved protocols (SYXK-2024).

### Statistical analysis

All datas are expressed as the means ± standard deviations (SD). Statistical analyses were performed using GraphPad Prism 9 software. P<0.05 was considered statistically significant.

## Results

### PDCoV infection downregulates CDC4 expression

**Figure 1.**
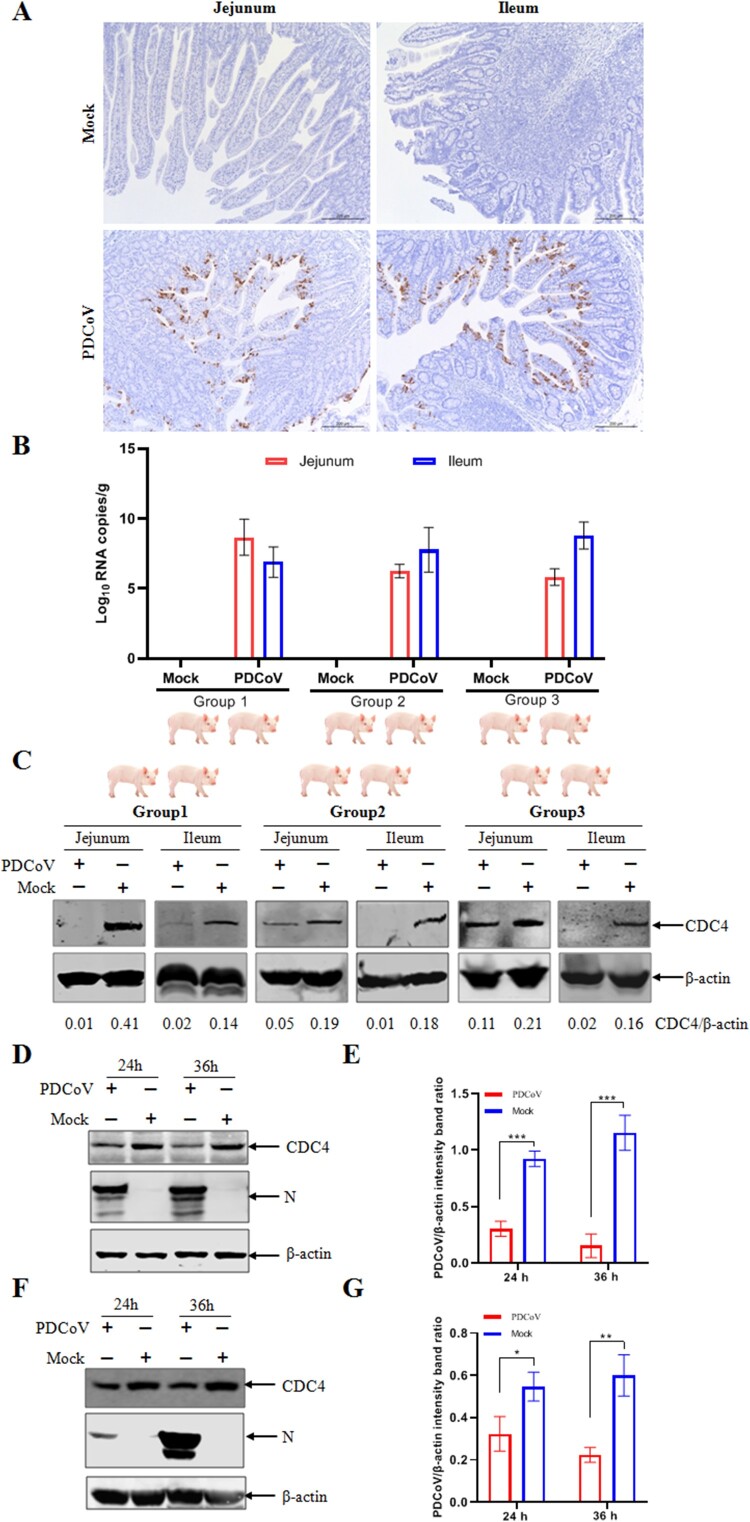
PDCoV infection downregulates CDC4 expression *in vivo.* (A) Evaluation of PDCoV infection in intestinal tissues from PDCoV-infected pigs by immunohistochemistry (IHC) (Scale bars, 200 μm). IHC detection of PDCoV antigens was performed by using the monoclonal antibody against PDCoV N protein, followed by an incubation with horseradish peroxidase (HRP)-conjugated sheep anti-mouse IgG. PDCoV antigen was detected by incubating with monoclonal antibody against PDCoV N protein. (B) Detection of PDCoV N mRNA in different intestinal tissues. The abundance of the mRNA of PDCoV N gene was determined by RT-qPCR. (C) Reduction of endogenous CDC4 followed by PDCoV infection. The expression of endogenous CDC4 was detected in jejunum and ileum in each group (challenge and control) by western blotting. Densitometric data of CDC4/β-actin was calculated at the bottom of the figure. (D and E) Downregulated expresssions of endogenous CDC4 post PDCoV infection in IPI-2I (D) and ST cells (E). Cells were infected with PDCoV at an MOI of 0.1 (IPI-2I) or 0.05 (ST), followed by CDC4 detection at indicated time points by western blotting. Densitometric data of CDC4/β-actin from three independent experiments are expressed as means ± SD. *P < .05, **P < .01, ***P < .001. The *P* value was calculated using Student’s *t*-tests.

During our research, animal experiments were carried out with PDCoV challenge and viral infection was confirmed when the virus staining and abundance of the mRNA of the PDCoV N gene were determined by immunohistochemistry (IHC) and quantitative real-time PCR (RT-qPCR), respectively. Compared to the mock-infected piglet, the IHC results indicated that severe atrophic enteritis was clearly observed in the jejunum and ileum of PDCoV-infected piglet (Figure 1(A)). Consistent with the intestinal lesion, high copies of PDCoV N mRNA were detected in intestinal tissues (jejunum and ileum) by RT-qPCR assay (Figure 1(B)). The reduction of endogenous CDC4 was apparently observed in the jejunum and ileum of PDCoV-infected piglets (Figure 1(C)). Next, we further examine whether PDCoV infection can reduce the expression of CDC4 in susceptible cell lines. IPI-2I cells and ST cells have been confirmed susceptible to PDCoV infection and widely used as essential models to isolate and investigate the pathogenic mechanisms of PDCoV infection *in vitro* [3]. As shown in Figure 1(D–G), the expression of CDC4 was significantly downregulated at indicated time points post PDCoV infection in both IPI-2I and ST cells, respectively. These results demonstrate that PDCoV infection induces CDC4 downregulations, hinting a regulatory role of CDC4 in PDCoV infection.

### CDC4 inhibits PDCoV infection

**Figure 2.**
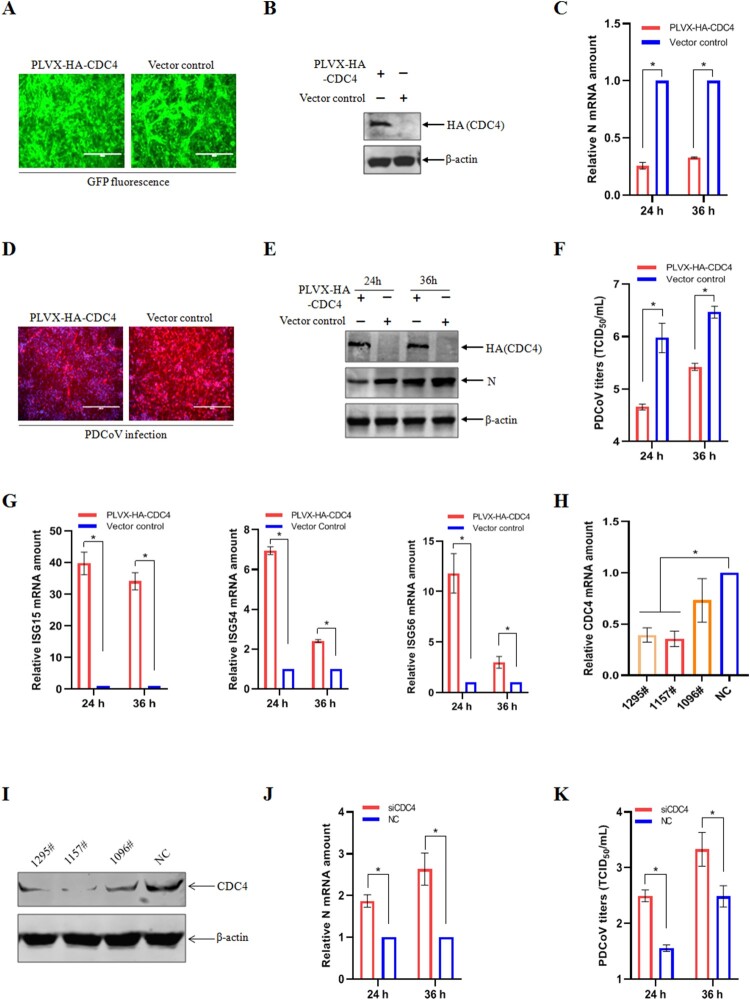
CDC4 inhibits PDCoV infection. (A) IPI-2I cells were inoculated with recombinant lentivirus containing CDC4 and ZsGreen or ZsGreen alone (vector control) as described in Materials and Methods. (B) Ectopic expression of recombinant lentivirus containing CDC4. IPI-2I cells were transduced with CDC4 or the vector control for 24 h, and cell samples were subjected to immunoblotting with indicated antibodies. (C to F) Overexpression of CDC4 suppressed PDCoV replication. IPI-2I cells were transduced with CDC4 or the vector control for 24 h and then inoculated with PDCoV at an MOI of 0.1 for further culture. PDCoV replication was evaluated by RT-qPCR (C), western blotting (D), IFA (E) and TCID_50_ (F), respectively. (G) CDC4 promoted the expressions of ISGs. IPI-2I cells were transduced with CDC4 or the vector control as described above and inoculated with PDCoV for another 24 h. The relative mRNA expressions of ISG15, ISG54 and ISG56 were determined by RT-qPCR. (H and I) Evaluation of knockdown efficiency of CDC4 siRNAs. Three siRNAs targeting CDC4 (siCDC4) were synthesized and referenced as 1295#, 1157# and 1096#, respectively. IPI-2I cells were transfected with negative control (NC) siRNA or siCDC4 for 24 h, followed by the determination of CDC4 transcription and expression by RT-qPCR (H) and western blotting analysis (I), respectively. (J and K) Knockdown of CDC4 facilitated PDCoV infection. IPI-2I cells were transfected with siRNAs and NC as described above and then infected with PDCoV (MOI  = 0.1) for another 24 and 36 h culture. The levels of PDCoV replication were detected by RT-qPCR (J) and TCID_50_ (K), respectively. The values are means from three independent infections (means ± SD). *P < .05. The *P*-value was calculated using Student’ s *t*-tests.

Previous studies have showed that CDC4 plays an important role in inhibitory replication of HCV, IAV, H1N1 and respiratory syncytial virus (RSV) [28,29,31]. IPI-2I cell line was originated from porcine intestinal epithelial cells and was recognized as an ideal model to investigate the pathogenic mechanisms of PDCoV infection *in vitro*. To explore the possible role of CDC4 in PDCoV infection, we investigated the regulatory effects of ectopically expressed CDC4 on the replication of PDCoV in IPI-2I cells. IPI-2I cells were inoculated with recombinant lentivirus containing N-terminal HA-tagged CDC4 and ZsGreen (green fluorescent protein), the lentivirus encoding ZsGreen alone served as a vector control. As shown in Figure 2(A), more than 90% of the cells were green fluorescence positive, which indicated that the lentiviruses were successfully transduced into IPI-2I cells. The ectopic expression of CDC4 was confirmed by western blotting in IPI-2I cells (Figure 2(B)). It was demonstrated that the mRNA levels of PDCoV N were decreased in the treated IPI-2I cells with CDC4 overexpression by RT-qPCR (Figure 2(C)). In addition, the levels of PDCoV N protein were significantly decreased in CDC4-overexpressing IPI-2I cells than that in vector control-treated parental cells by IFA (Figure 2(D)). Consistent with the inhibitory effects of viral protein expression, overexpression of CDC4 resulted in remarkable inhibition of PDCoV replication by western blotting (Figure 2(E)). Moreover, a lower viral titre was observed in CDC4-overexpressing IPI-2I cells relative to the mock cells by the 50% tissue culture infective dose (TCID_50_) (Figure 2(F)). Similarly, a decrease in PDCoV replication was further confirmed followed by ectopic expressions of CDC4 in ST cells (Figure S1). Subsequently, we examined whether the expression of CDC4 could induce the positive activation of ISGs. When target cells were transfected with CDC4 plasmid prior to PDCoV infection, the transcriptional levels of innate antiviral molecules, such as ISG15, ISG54 and ISG56, were calculated by RT-qPCR. As expected, the ectopic expression of CDC4 resulted in the increased transcriptional levels of ISGs in PDCoV infected IPI-2I cells (Figure 2(G)). Collectively, these results indicate that CDC4 plays an antiviral role in PDCoV replication.

To further confirm the effect of CDC4 on PDCoV infection, three short interfering RNAs (siRNAs) targeting CDC4 (siCDC4-1295#, 1157# and 1096#) were designed and synthesized to evaluate the role of CDC4 knockdown in regulation of PDCoV replication. The levels of CDC4 mRNA (Figure 2(H)) and protein (Figure 2(I)) were more significantly downregulated in IPI-2I cells transfected with siCDC4-1157# by RT-qPCR and western blotting analysis, respectively, compared to the relatively high levels of CDC4 expression in the negative control (NC) siRNA-transfected cells. Subsequently, IPI-2I cells were transfected with siCDC4-1157# for 24 h and then infected with PDCoV at an MOI of 0.1, followed by determination of viral replication by RT-qPCR and TCID_50_ methods. It was shown that the levels of PDCoV N mRNA were elevated at 24 and 36 h in CDC4 knockdown IPI-2I cells, respectively, compared to that in the mock cells (Figure 2(J)). Consistently, a higher viral titre was confirmed in IPI-2I cells transfected with siCDC4-1157# than that in IPI-2I cells transfected with siRNA NC (Figure 2(K)). Taken together, these results suggest that knockdown of endogenous CDC4 enhances the replication of PDCoV in IPI-2I cells.

### Nuclear shuttling of CDC4 is critical for the antiviral activity

**Figure 3.**
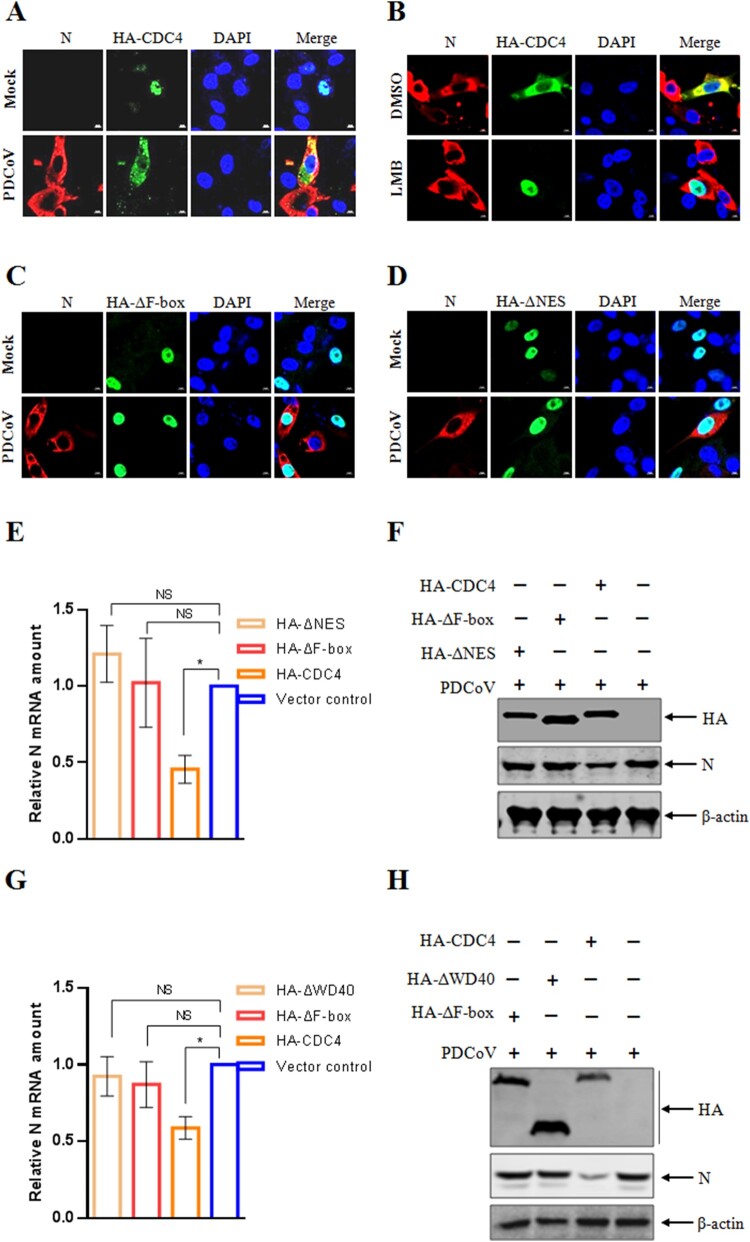
Nuclear shuttling of CDC4 is critical for its antiviral activity. (A)PDCoV infection mediated CDC4 translocation from the nucleus into the cytoplasm. IPI-2I cells were transfected with HA-CDC4 for 24 h and then infected with PDCoV (MOI  = 0.1). Cell monolayers were fixed at 24 h post infection, and triple-color immunofluorescence staining was performed to detect the CDC4 (green), and PDCoV N protein (red) and the nucleus (blue). The fluorescence was observed by a confocal laser scanning microscope (Zeiss). (B) The cytoplasmic localization of CDC4 was blocked by treatment with nuclear export inhibitor of LMB. IPI-2I cells were transfected with HA-CDC4 followed by PDCoV inoculation for 24 h. The cells were treated with LMB (2.5 nM) or carrier control (DMSO) for 6 h before the samples were collected to detect the CDC4 (green), and PDCoV N protein (red) and the nucleus (blue) by immunofluorescence staining. (C and D) F-box and NES are crucial for CDC4 translocation in the context of PDCoV infection. IPI-2I cells were transfected with HA-CDC4 mutants carrying the deletion of F-box (HA-ΔF-box) (C) or NES mutations (HA-ΔNES) (Isoleucine at position 281 and leucine at positions 284, 288 and 290 were all replaced with glycine) (D) for 24 h and then infected with PDCoV (MOI = 0.1). As previously described, the fluorescence staining was performed to detect the CDC4 (green), and PDCoV N protein (red) and the nucleus (blue) at 24 h post infection. (E and F) F-box and NES are required for the antiviral activity of CDC4. IPI-2I cells were transfected with HA-CDC4, HA-ΔF-box, HA-ΔNES or vector control for 24 h, respectively, followed by PDCoV inoculation for another 24 h. The levels of PDCoV replication were determined by RT-qPCR (E) and western blotting analysis (F).(G and H) Intact CDC4 was critical to exert antiviral function. IPI-2I cells were transfected with HA-CDC4, HA-ΔF-box, HA-CDC4 mutants carrying the deletion of WD40 domain (HA-ΔWD40) or vector control for 24 h, respectively, followed by PDCoV inoculation for another 24 h. PDCoV replication was evaluated by RT-qPCR (G) and western blotting (H). The values are means from three independent infections (means ± SD). *P < .05. The *P*-value was calculated using Student’ s *t*-tests.

Protein subcellular localization is tightly controlled and intimately linked to protein function in diverse statuses of cell biology, such as health, disease and virus infection. It remains unclear whether the intracellular distribution of CDC4 might be changed in the context of PDCoV infection. Surprisingly, nuclear-cytoplasmic shuttling of CDC4 was detected in the PDCoV infected IPI-2I cells (Figure 3(A)). We next questioned that whether the nuclear export signal (NES) was involved in the nuclear shuttling of CDC4 during PDCoV infection. CDC4-overexpressed IPI-2I cells were subject to PDCoV infection and treated with leptomycin B (LMB), a specific nuclear export inhibitor [32], during the whole infection process. As shown in Figure 3(B), the translocation from nucleus to the cytoplasm of CDC4 was totally blocked by LMB, indicating that the critical role of nuclear export signal in the nuclear-cytoplasmic shuttling of CDC4 in PDCoV infected IPI-2I cells. In general, nuclear-cytoplasmic shuttling proteins usually contain NES or sequences, which is directly recognized and bound by exportins, such as chromosome region maintenance 1 (CRM1). A consensus NES has been reported to be shared at the start of the F-box domain among the members in the family, including Fbxo2/NFB42, Fbxo4, Fbxo6, Fbxo7, Fbxo8, Fbxl5, Fbxw2 and CDC4 [33]. To identify the NES domain(s) in CDC4 necessary for its cytoplasmic localization, IPI-2I cells were transfected with HA-tagged mutants of CDC4 with deletion of F-box domain (ΔF-box) followed by PDCoV infection to evaluate the intracellular localization of CDC4. The confocal assay revealed that the nuclear-cytoplasmic shuttling of CDC4 was inhibited during PDCoV infection due to the loss of NES located at F-box domain (Figure 3(C)). According to the characterized NES consensus sequence [34], we constructed the CDC4 NES mutant of which isoleucine at position 281 and leucines at positions of 284, 288 and 290 were all replaced with glycine (ΔNES), and then the CDC4 ΔNES mutant was transfected into IPI-2I cells followed by PDCoV inoculation. Consistent with the results from the deletion of F-box domain, function of NES loss abolished the nuclear-cytoplasmic shuttling of CDC4 during PDCoV infection (Figure 3(D)). These data suggested that NES-CRM1 pathway was involved in PDCoV infection mediated nuclear-cytoplasmic shuttling of CDC4. However, whether nuclear-cytoplasmic shuttling is related to the antiviral activity of CDC4 remains unclear. To investigate the participation of CDC4 shuttling in regulation of PDCoV replication, IPI-2I cells were transfected with WT and mutation plasmids for 24 h, respectively, and then infected with PDCoV at an MOI of 0.1 followed by determination of PDCoV infection by RT-qPCR and western blotting at indicated time points. Compared with the inhibitory activity of WT CDC4, the antiviral activity was deprived in IPI-2I cells transfected with ΔF-box or ΔNES mutant of CDC4 when determined by RT-qPCR (Figure 3(E)) and western blotting analysis (Figure 3(F)), respectively. In addition, we further evaluated the involvement of WD40 domain in CDC4 mediated antiviral activity. The antiviral effects were lost due to the deletion of WD40 domain as determined by RT-qPCR (Figure 3(G)) and western blotting analysis (Figure 3(H)), suggesting the requirement of intact CDC4 to inhibit PDCoV replication. The mentioned results indicated that nuclear shuttling plays an important role in the antiviral activity of CDC4 in the context of PDCoV infection.

### Involvement of N protein in nuclear-cytoplasmic shuttling of CDC4

**Figure 4.**
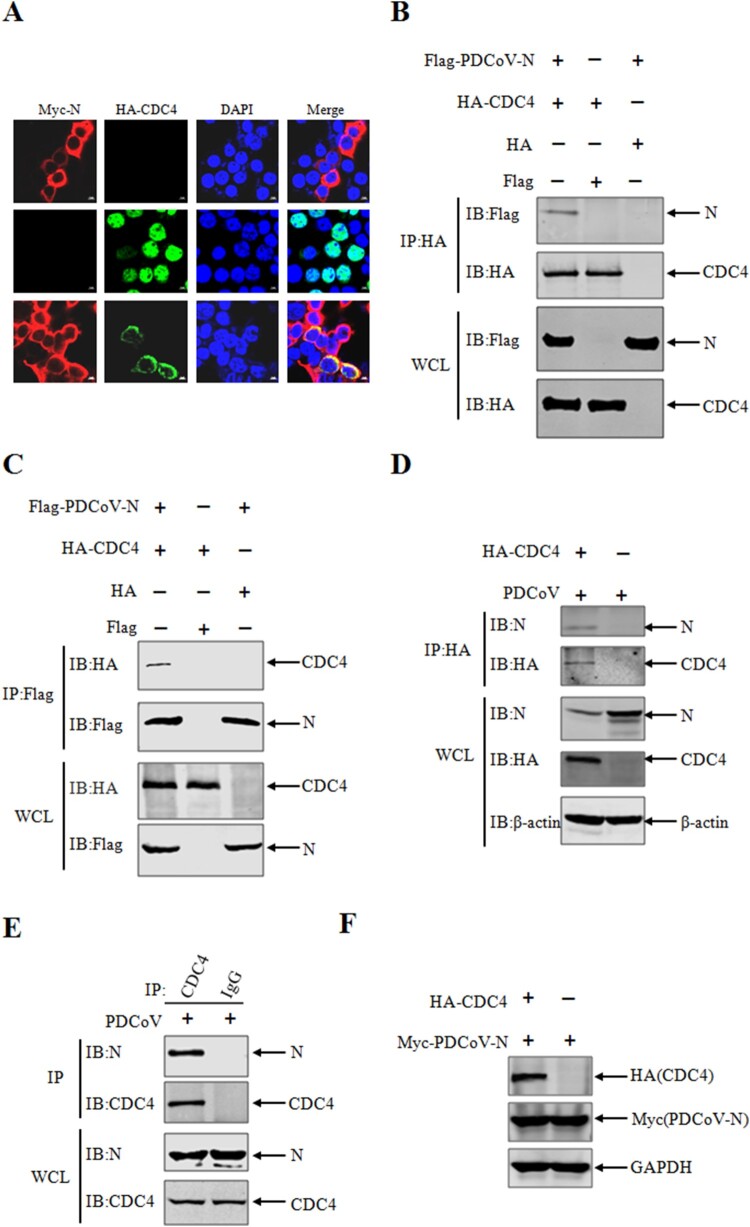
CDC4 interacts with PDCoV N protein. (A) Involvement of N protein in the CDC4 shuttling and colocalization between PDCoV N protein and CDC4. HEK 293 T cells were co-transfected with HA-CDC4 and Myc-PDCoV-N, and transfected with HA-CDC4 or Myc-PDCoV-N alone as controls. The cells were stained to detect PDCoV N protein (red), CDC4 (green) and the nucleus (blue) by confocal microscopy. (B and C) Identification of interactions between CDC4 and PDCoV N. HEK 293 T cells were co-transfected with HA-CDC4 and Flag-PDCoV-N, and transfection of HA-CDC4 or Flag-PDCoV-N alone was left as controls. At 36 h post transfection, the cells were collected and lysed for Co-IP analysis with anti-HA (B) or anti-Flag (C) antibodies, respectively. (D) IPI-2I cells were transfected with HA-CDC4 for 24 h, and then infected with PDCoV (MOI = 0.1) for another 24 h culture. The Co-IP analysis was performed with anti-HA antibody as indicated. (E) IPI-2I cells were infected with PDCoV (MOI  = 0.1), followed by Co-IP analysis with anti-CDC4 antibody at 24 h post infection. (F) The effect of CDC4 on the expression of PDCoV N protein. HEK 293 T cells were co-transfected with HA-CDC4 and Myc-PDCoV-N for 36 h, and were harvested for western blotting analysis.

It has been confirmed by the previous data that PDCoV infection contributed to the nuclear-cytoplasmic shuttling of CDC4 (Figure 3(A)). We further questioned that which viral encoded protein was involved in the translocation of CDC4. Based on the previous observations, the staining of N protein was well localized with CDC4 in the cytoplasm as determined by confocal assay post PDCoV infection (Figure 3(A-B)), speculating that viral N protein might interact and target CDC4 for translocation from nucleus into cytoplasm. As expected, the nuclear distribution of CDC4 was markedly altered when Myc-PDCoV-N was coexpressed in target cells, which suggested that the N protein of PDCoV is crucial to the CDC4 shuttling from nuclear to cytoplasmic distribution (Figure 4(A)). To further confirm the interactions, the Co-IP assay was performed as described previously [35]. HEK 293 T cells were co-transfected with Flag-tagged PDCoV N plasmids and HA-tagged CDC4 or empty vector control for 24 h followed by Co-IP assay with anti-HA antibody. It was corroborated that there was an interaction between PDCoV N and CDC4 (Figure 4(B)). And vice versa, PDCoV N proteins were detected by the reverse Co-IP assay with anti-Flag antibody (Figure 4(C)), suggesting the existence of the interaction between CDC4 and PDCoV N protein *in vitro*. Additionally, PDCoV N protein can be pulled down by ectopically expressed CDC4 (HA-tagged) in the context of PDCoV infection (Figure 4(D)). More importantly, the endogenous interaction between CDC4 and PDCoV N protein was also confirmed in PDCoV infected IPI-2I cells (Figure 4(E)). As well known that CDC4 is determined as an E3 ubiquitin ligase to degrade diverse substrates [36], we thus questioned that whether CDC4 could also induce the degradation of PDCoV N protein. Objectively, CDC4 failed to reduce the expression of PDCoV N protein (Figure 4(F)). In summary, the data proved that there were interactions between CDC4 and PDCoV N protein, and PDCoV N protein was involved in CDC4 shuttling in the context of PDCoV infection.

### CDC4 counteracts the inhibitory effect of PDCoV N on type I IFN production

**Figure 5.**
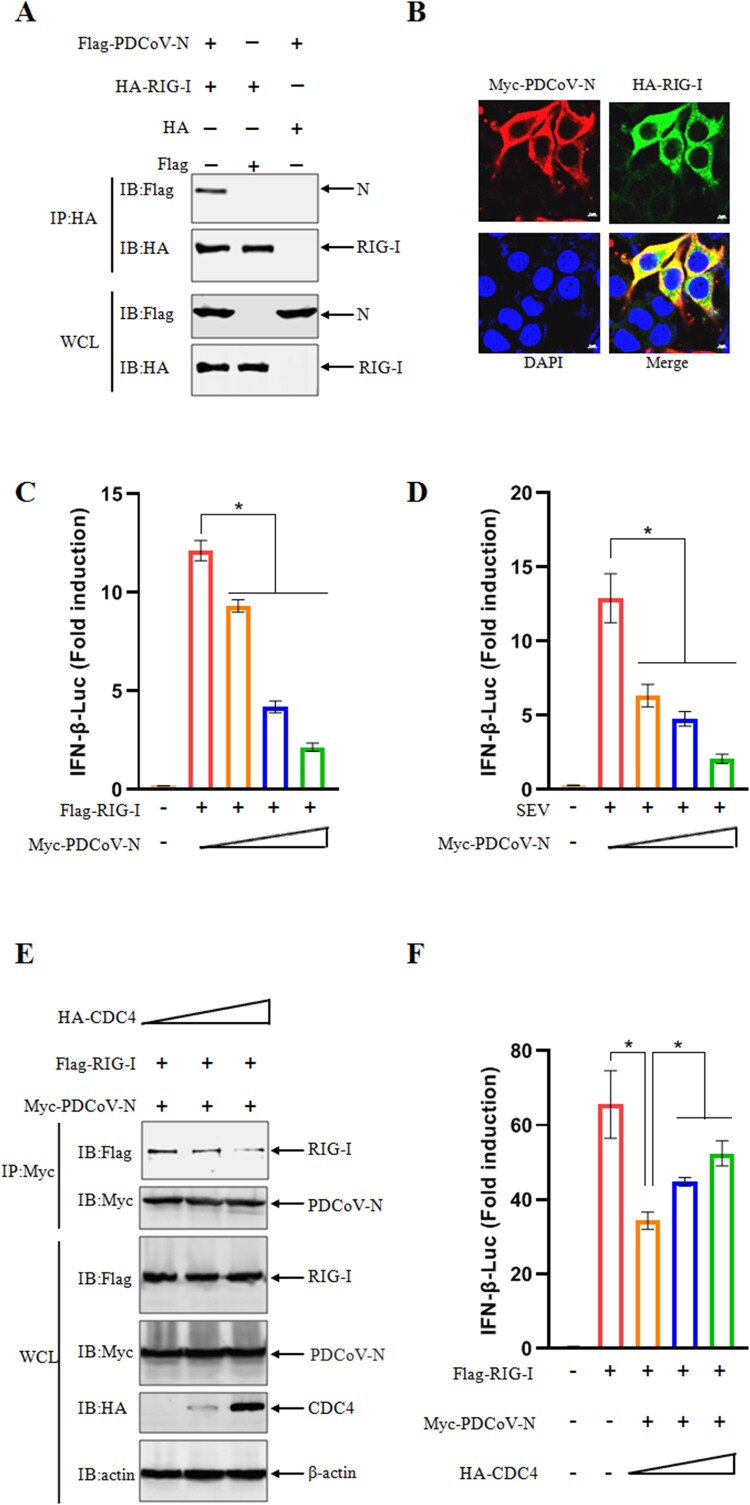
CDC4 counteracts the inhibitory effect of PDCoV N on type I IFN production. (A) PDCoV N protein interacted with RIG-I by Co-IP. HEK 293 T cells were transfected with Flag-PDCoV-N and HA-RIG-I, and the cells were lysed and subjected to Co-IP with anti-HA antibody. (B) Identification of interactions between PDCoV N and RIG-I by confocal microscopy. HEK 293 T cells were co-transfected with Myc-tagged PDCoV N and HA-tagged RIG-I followed by staining with indicate antibodies at 36 h. The fluorescent signals were observed with confocal microscope. (C) PDCoV N protein suppresses RIG-I -induced IFN-β production. HEK 293 T cells were co-transfected with IFN-β-Luc (0.15 μg), pRL-TK (0.015 μg) and Flag-RIG-I plasmids (0.1 μg), along with the increasing amount of Myc-PDCoV-N plasmids (0, 0.2, 0.4 or 0.6 μg). Dual-luciferase reporter assays were performed to evaluate the activation of the IFN-β promoter at 24 h post transfection. (D) PDCoV N protein antagonizes SEV-induced IFN-β production. HEK 293 T cells were co-transfected with IFN-β–Luc (0.15 μg), pRL-TK (0.015 μg) and the increasing amount of Myc-PDCoV-N plasmids (0, 0.2, 0.4 or 0.6 μg) for 24 h, followed by inoculation with SEV for another 12 h. The activation of the IFN-β promoter was determined by dual-luciferase reporter assays. (E) CDC4 disrupts the interaction of PDCoV N and RIG-I. HEK 293 T cells were co-transfected with Flag-RIG-I, Myc-PDCoV-N and the increasing amounts of HA-CDC4 for 36 h, followed by Co-IP assays with anti-Myc antibody. (F) CDC4 alleviates Myc-PDCoV-N mediated antagonism of antiviral response. Plasmids of IFN-β-Luc (0.15 μg), pRL-TK (0.015 μg), Flag-RIG-I (0.1 μg) and Myc-PDCoV-N (0.3 μg), together with the increasing amount of HA-CDC4 (0, 0.3 or 0.6 μg) were transfected into HEK 293 T cells for 24 h. The activation of the IFN-β promoter was determined by dual-luciferase reporter assays. The values are means from three independent infections (mean ± SD). *P < .05. The *P*-value was calculated using Student’ s *t*-tests.

To further dissect the detailed biological activity of PDCoV N protein, a series of identifications have been performed to screen the specific targets potentially interacted with PDCoV N. When immunoprecipiated with the key adaptors (RIG-I, MAVS, TBK1 and IRF3) of innate immune signalling pathway (Figure S2), PDCoV N was confirmed to interact with RIG-I protein (Figure 5(A, B)). Based on the interaction of PDCoV N protein with RIG-I, a dual-luciferase reporter assay was carried out to investigate the regulatory effects of PDCoV N protein on IFN-β production. As shown in Figure 5(C), RIG-I significantly induced the activation of the IFN-β-Luc promoter, but the activation levels were impaired as the abundance of ectopic expressions of PDCoV N protein were gradually increased in target cells. Furthermore, we checked the inhibitory effects of PDCoV N protein on the activation of IFN-β-Luc promoters in the context of SeV infection. Similarly, overexpression of PDCoV N protein disrupted the SEV-induced activation of IFN-β-Luc promoters in a dose dependent manner (Figure 5(D)). These results indicate that PDCoV N protein antagonizes IFN-β production by competitive binding with RIG-I.

Our previous data have demonstrated that CDC4 exerts obvious antiviral activity against PDCoV infection. However, the detailed antiviral mechanism of CDC4 to inhibit PDCoV replication remains unclear. Increasing studies have identified that CoVs N proteins were widely involved in antagonisms of the host innate immune defense [37-39]. Based on the confirmed interactions of PDCoV N with RIG-I and CDC4 in our study, it is reasonable to speculate that CDC4 could exert the antiviral activity by competing with RIG-I to bind PDCoV N protein. To test this hypothesis, HEK 293 T cells were co-transfected with Flag-RIG-I and Myc-PDCoV N along with increasing amounts of HA-CDC4 or vector control, followed by determination of interactions between Flag-RIG-I and Myc-PDCoV-N by Co-IP assays. As shown in Figure 5(E), RIG-I was pulled down by PDCoV N, and however, the amount of RIG-I immunoprecipitated by PDCoV N was decreased as the abundance of ectopic CDC4 protein was increased, suggesting that CDC4 impaired the interaction between RIG-I and PDCoV N. Additionally, a dual-luciferase reporter assay was performed to explore the role of CDC4 in the antagonisms of PDCoV N inhibiting RIG-I activated signalling response. The antagonistic activity of PDCoV N protein was obviously decreased as the increasing dose of CDC4 was transfected in target cells (Figure 5(F)). Therefore, the results concluded that CDC4 plays a positive role in antagonizing the inhibitory effect of PDCoV N on type I IFN production.

### CDC4 mediates antiviral activity by the competitive binding of TGEV N protein with RIG-I

**Figure 6.**
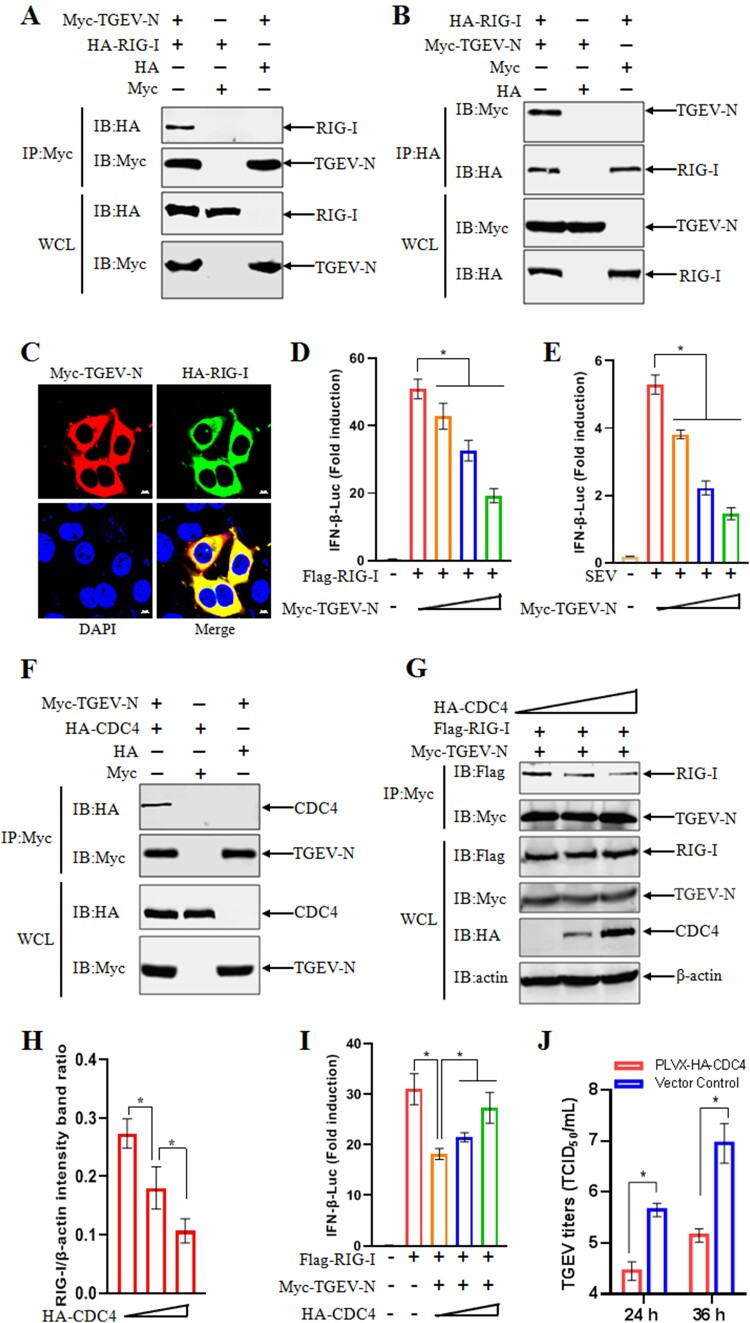
CDC4 exerts antiviral activity by the competitive binding of TGEV N protein with RIG-I. (A and B) TGEV N protein interacts with RIG-I. HEK 293 T cells were transiently transfected with Myc-TGEV-N and HA-RIG-I, followed by Co-IP analysis with anti-Myc (A) or anti-HA (B) antibodies. (C) Interactions of TGEV N with RIG-I by confocal microscopy. HEK 293 T cells were co-transfected with Myc-tagged TGEV N and HA-tagged RIG-I, followed by staining to detect TGEV N protein (red), RIG-I (green) and the nucleus (blue) by confocal microscopy. (D) TGEV N protein suppresses RIG-I-induced IFN-β production. HEK 293 T cells were co-transfected with IFN-β–Luc (0.15 μg), pRL-TK (0.015 μg) and Flag-RIG-I (0.1 μg) plasmids, along with the increasing amount of Myc-TGEV-N plasmids (0, 0.2, 0.4 or 0.6 μg). Dual-luciferase reporter assays were performed to evaluate the activation of the IFN-β promoter at 24 h post transfection. (E) TGEV N protein disrupts SEV-induced IFN-β production. IFN-β-Luc (0.15 μg) together with the pRL-TK (0.015 μg) plasmid and the increasing amount of Myc-TGEV-N (0, 0.2, 0.4 or 0.6 μg) were transfected into HEK 293 T cells for 24 h. Following by inoculation with SEV for another 12 h, the levels of IFN-β promoter activation were determined by dual-luciferase reporter assays. (F) TGEV N protein interacts with CDC4. Plasmids of Myc-TGEV-N and HA-CDC4 were co-transfected into HEK 293 T cells, followed by Co-IP assay with anti-Myc antibody at 36 h post transfections. (G and H) CDC4 disrupts the interactions between TGEV N and RIG-I. HEK 293 T cells were co-transfected with Flag-RIG-I, Myc-TGEV-N and the increasing amount of HA-CDC4 for 24 h, followed by Co-IP assays with anti-Myc antibody. Densitometc data of RIG-I/β-actin from three independent experiments are expressed as mean ± SD. (I) CDC4 counteracts the antagonism of antiviral response mediated by TGEV N proteins. Plasmids of IFN-β–Luc (0.15 μ), pRL-TK (0.015 μg), Flag-RIG-I (0.1 μg) and Myc-TGEV-N (0.3 μg), together with the increasing amount of HA-CDC4 (0, 0.3 or 0.6 μg) were transfected into HEK 293 T cells for 24 h. The activation of the IFN-β promoter was determined by dual-luciferase reporter assays. (J) Overexpression of CDC4 inhibits TGEV replication in IPI-2I cells. IPI-2I cells were transduced with CDC4 or the vector control for 24 h, and then inoculated with TGEV (MOI = 0.5) for further culture. At 24 and 36 h post infection, the level of TGEV replication was evaluated by TCID_50_. The values are means from three independent infections (means ± SD). *P < .05. The *P*-value was calculated using Student’ s *t*-tests.

Next, we further asked whether the similar biological role of CDC4 was observed in the context of TGEV infection. As expected, the interaction between TGEV N and RIG-I was confirmed by Co-IP experiment (Figure 6(A, B)) and confocal assay (Figure 6(C)). Due to the interaction with RIG-I, it was clearly shown that TGEV N clearly inhibits the RIG-I activated signalling response in a dose dependent manner (Figure 6(D)). In addition, the regulatory effects of TGEV N on IFN-β production were also evaluated in the context of SEV infection. As shown in Figure 6(E), the activation of IFN-β-Luc promoter was apparently alleviated by the increasing transfections of ectopic TGEV N protein, implying the antagonistic effects of TGEV N on innate antiviral response. Next, the existence of interaction was proved between CDC4 and TGEV N when determined by Co-IP assay (Figure 6(F)). Importantly, RIG-I was confirmed to be immunoprecipitated by TGEV N, but the levels of RIG-I protein pulled down by TGEV N was gradually reduced as the expression of CDC4 protein was increased, which demonstrated that CDC4 disturbed the interaction between RIG-I and TGEV N (Figure 6(G) and (H)). Consistent with the positive role of CDC4 as mentioned above, the RIG-I induced IFN-β-Luc activity was significantly suppressed by the expression of TGEV N proteins, and the decrease in IFN-β-Luc activation was markedly compromised by the addition of CDC4 transfections (Figure 6(I)). Subsequently, the antiviral effects of CDC4 were determined by ectopic expression of CDC4 followed by TGEV infection in IPI-2I cells. The ectopic expression of CDC4 induced the elevated levels of ISGs in TGEV infected IPI-2I cells (Figure S3). As expected, the replication of TGEV was obviously inhibited by CDC4 when the titre was determined by TCID_50_ (Figure 6(J)). Same as the antiviral role in IPI-2I cells, overexpression of CDC4 resulted in apparent inhibition of TGEV infection in ST cells (Figure S4).

### Shared antiviral mechanism of CDC4 against SARS-CoV-2

**Figure 7.**
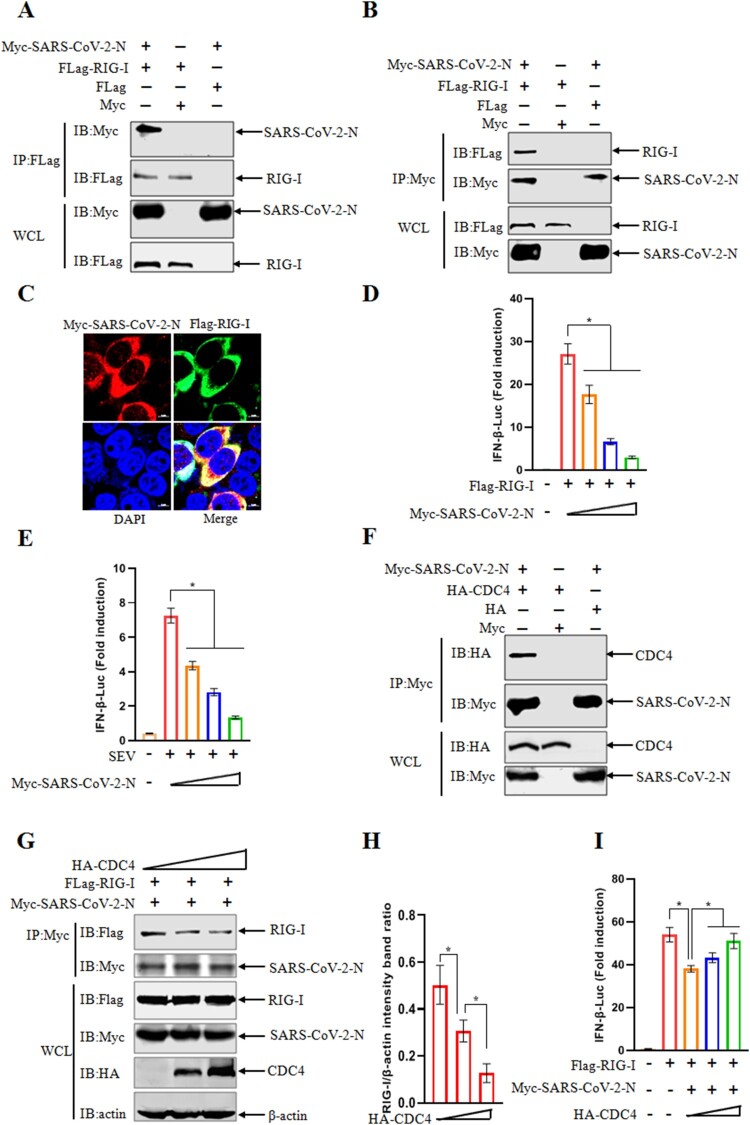
Shared antiviral mechanism of CDC4 against SARS-CoV-2. (A and B) SARS-CoV-2-N protein interacts with RIG-I. HEK 293 T cells were transfected with Myc-SARS-CoV-2-N and Flag-RIG-I, followed by Co-IP analysis with anti-Flag (A) or anti-Myc (B) antibodies. (C) Confirmation of interactions between SARS-CoV-2-N and RIG-I by confocal microscopy. HEK 293 T cells were co-transfected with Myc-SARS-CoV-2-N and Flag-RIG-I, followed by staining to detect SARS-CoV-2-N protein (red), RIG-I (green) and the nucleus (blue) by confocal microscopy. (D) SARS-CoV-2-N protein disrupts RIG-I-induced IFN-β production. HEK 293 T cells were co-transfected with IFN-β–Luc (0.15 μg), pRL-TK (0.015 μg) and Flag-RIG-I (0.1 μg) plasmids, along with the increasing amount of Myc-SARS-CoV-2-N plasmids (0, 0.2, 0.4 or 0.6 μg). The activation of the IFN-β promoter was analysed by the dual-luciferase reporter assay at 24 h post transfection. (E) SARS-CoV-2-N protein restrains SEV-induced IFN-β production. HEK 293 T cells were transfected with IFN-β-Luc (0.15 μg) and pRL-TK (0.015 μg) plasmids along with the increasing amount of Myc-SARS-CoV-2-N (0, 0.2, 0.4 or 0.6 μg) for 24 h. When the cells were inoculated with SEV for another 12 h, the level of IFN-β promoter activation was determined by the dual-luciferase reporter assay. (F) SARS-CoV-2- N protein interacts with CDC4. HEK 293 T cells were co-transfected with plasmids of Myc-SARS-CoV-2-N and HA-CDC4, followed by Co-IP assay with anti-Myc antibody at 36 h post transfections. (G and H) CDC4 competes the interaction between SARS-CoV-2-N and RIG-I. HEK 293 T cells were co-transfected with Flag-RIG-I, Myc-SARS-CoV-2-N and increasing amounts of HA-CDC4 for 36 h, followed by Co-IP assays with anti-Myc antibody. Densitometric data of RIG-I/β-actin from three independent experiments are expressed as means ± SD. (I) CDC4 counteracts the antagonism of antiviral response mediated by SARS-CoV-2-N protein. Plasmids of IFN-β–Luc (0.15 μg), pRL-TK (0.015 μg), Flag-RIG-I (0.1 μg) and SARS-CoV-2-N (0.3 μg), together with the increasing amount of HA-CDC4 (0, 0.3 or 0.6 μg) were transfected into HEK 293 T cells for 24 h. The activation of the IFN-β promoter was determined by the dual-luciferase reporter assay. The values are means from three independent infections (mean ± SD). *P < .05. The *P*-value was calculated using Student’s *t*-tests.

CoVs are a diverse family of viruses that infect a wide host range, including avian and mammalian. In the twenty-first century, CoVs have frequently broadened their host range, with three events resulting in severe disease outbreaks in human populations. Three highly pathogenic coronaviruses with zoonotic origin, severe acute respiratory syndrome coronavirus (SARS-CoV), Middle East respiratory syndrome coronavirus (MERS-CoV) and SARS-CoV-2, emerged in humans and caused fatal respiratory illness in 2002, 2012 and 2019, respectively [40-42], making the emerging coronavirus as a new public health concern in the future. Being highly transmissible, this novel coronavirus disease, known as coronavirus disease 2019 (COVID-19), has spread fast all over the world. It has overwhelmingly surpassed SARS and MERS in terms of both the number of infected people and the spatial range of epidemic areas. However, the ongoing outbreak of COVID-19 has posed an extraordinary threat to global public health in the past few years [43]. Here, we questioned that whether the similar role of CDC4 was observed in the biological process of SARS-CoV-2 infection. Similarly, the interaction between SARS-CoV-2-N and RIG-I was detected by Co-IP assays (Figure 7(A, B)), which was corroborated by the confocal assay (Figure 7(C)). When the relative activity of IFN-β-Luc promoter was calculated by dual-luciferase reporter assay, SARS-CoV-2-N was proved to inhibit the IFN-β production activated by RIG-I transfection or SEV infection (Figure 7(D, E)). Similar with the role of CDC4 in PDCoV or TGEV infection, CDC4 was identified to interact with SARS-CoV-2-N (Figure 7(F)) and disrupted the binding of SARS-CoV-2-N with RIG-I in a dose dependent manner (Figure 7(G) and (H)). Based on the data of CDC4 to block the interaction between SARS-CoV-2-N and RIG-I, we further investigate the regulatory effects of CDC4 on the SARS-CoV-2-N mediated suppression in RIG-I induced signalling pathway. Not surprisingly, the antagonistic activity of SARS-CoV-2-N was obviously impeded in HEK 293 T cells treated with addition of CDC4 (Figure 7(I)). Collectively, the results demonstrated that CDC4 might play an important role in controlling the replication of diverse coronaviruses from different Coronavirus genus, such as PDCoV, TGEV and SARS-CoV-2. A shared antiviral mechanism of CDC4 was unveiled that CDC4 inhibits the infection of diverse CoVs by blocking the antagonism of CoVs N proteins on RIG-I associated signalling pathway.

## Discussion

CDC4 participates in variant activities of biological processes, and is crucial for recognizing and binding to substrates. Emerging evidence exhibits that CDC4 has been confirmed as a tumour suppressor due to the wide involvements in degradation of diverse proteins associated with oncogenic developments, including cyclin E, c-Myc, c-Myb, c-Jun, Notch, MCL1, MED13/13L, PGC-1α, C/EBPα, TGIF1 and KLF5 [22-27]. But the literature on the interaction between CDC4 and virus infection remains less clear. Increasing evidence has shown that CDC4 plays an important role in suppressing the replication of several viruses, including IAV, HCV, H1N1 and RSV [28,29,31]. Although the antiviral capacity of CDC4 was verified by the previous research, the antagonism derived from virus infection has also been proved by several investigations. Primary effusion lymphoma (PEL) is an aggressive B cell lymphoma that is etiologically linked to Kaposi’s sarcoma-associated herpesvirus (KSHV). KSHV encoded latency-associated nuclear antigen (LANA) competes with the anti-apoptotic protein MCL-1 to bind with E3 ubiquitin ligase CDC4, leading to the increased stability of MCL-1 by preventing its proteasome-mediated degradation. The caspase-3-mediated apoptosis was inhibited by the enhanced abundance of MCL-1, which facilitates the tumorigenesis of KSHV + BCBL-1 cells/KSHV + PEL [44]. Isobe et.al reported that the oncogene product of E1A derived from adenovirus interacts with SCF(CDC4) and can attenuate the ubiquitylation of its target proteins so as to interfere with the activity of the SCF (CDC4) ubiquitin ligase [45]. The CCNE2, as the substrate of CDC4 was reported to act as a dependency factor to induce HIV Tat activity by encoding protein Cyclin E1, Cdk2 and Cyclin E. It is speculated that HIV-1 hijacks CDC4 to sequester it to prevent the degradation of CCNE2, facilitating HIV-1 infection in the target cells [46]. In the present study, we have demonstrated that CDC4 can translocate from the nucleus into the cytoplasm in the PDCoV-infected cells and PDCoV N was involved in the shuttling of CDC4 (Figure 4). Furthermore, it was confirmed that the cytoplasmic translocation of CDC4 plays a crucial role in the achievement of its antiviral function (Figure 3(E, F)). Collectively, the close associations between CDC4 and virus infection were observed and the detailed shuttling mechanisms retains to be further investigated in the future. Based on a series of experiments on the cellular levels, we reveal a novel function of CDC4 protein in restricting PDCoV infection in this study. Further investigations need to be designed and carried out about how to explore the feasibility of CDC4 as an antiviral target. As the fact, CDC4 was localized and exerted an antiviral role in the cells but not on the cellular surface or in the small intestinal mucus as the secreted protein. Moreover, oral administration of exogenous CDC4 could result in degradation of CDC4 by digestive enzymes in the stomach. Therefore, oral administration of exogenous CDC4 proteins could not provide effective protections against PDCoV infection. Instead, we can choose indirect ways to increase the antiviral activities of host by inducing the increased expressions of endogenous CDC4, achieving the indirect antiviral applications of CDC4 in pig farms. On the one hand, we can screen the drugs targeting CDC4 related pathways to elevate the expression levels of CDC4. Besides, probiotics have been recognized as the commonly-used additive in animal feed, including *Bacillus subtilis* [47]. We could utilize probiotic platforms to design a series of *Bacillus subtilis* expressing target genes, and then screen the recombinant candidates that can induce the increased expressions of CDC4 in host cells post oral administration. After the protective assessment of potential candidates by challenge experiments, we might explore the feasibility of CDC4 as an antiviral target.

CoVs are a diverse group of viruses infecting many different hosts, and they can cause mild to severe diseases in humans and animals. SARS-CoV and MERS-CoV are first emerged in 2002 and 2012, respectively, and corroborated highly transmissible and pathogenic in humans to caused fatal respiratory illness, making coronaviruses as new public health concern worldwide. From late 2019, another novel coronavirus, named SARS-CoV-2 caused a pandemic of acute respiratory disease (COVID-19) with large-scale deaths and injuries and posed an extraordinary threat to global public health [43]. Similarly, some novel and variant coronaviruses (e.g. PDCoV, variant strain of PEDV, swine acute diarrhoea syndrome-coronavirus (SADS-CoV)) emerged and caused severe diarrhoea-associated diseases with great economic losses in the porcine industry all over the world [48]. As well known, IFN-mediated antiviral response is an important component of virus-host interactions and plays an essential role in inhibiting virus infection. The accumulating evidence documented that coronaviruses have evolved multiple strategies to evade the innate antiviral response of the host cells [49-51]. Similar to the other coronaviruses, PDCoV can effectively restrain the IFN-mediated innate antiviral response of target cells through diverse antagonistic strategies, including the degradation of crucial innate signalling regulators via the ubiquitin-proteasomal or autophagy pathway [52,53], cleavage of antiviral factors by the viral encoded protein, such as the 3C-like protease [49,54] and interference of the interactions of adaptor proteins and sensor recognition required for the initiation and/or activations of relative signalling pathways [55]. Among the viral encoded proteins, N protein is one of the most abundant structural proteins of CoVs, and was reported to play important roles in evading the host’s antiviral response [56]. For example, the N proteins of SARS-CoV and MHV interacted with protein activator of protein kinase R (PACT) to disrupt the initial step of RLR signalling pathway [39]. The N proteins of SADS-CoV succeeded to reduce IFN-β production by blocking the interaction between TRAF3 and TBK1 [57]. It was confirmed that SARS-CoV-2-N can antagonize the formation of stress granules to suppress IFN production [58,59]. Moreover, the MAVS SUMOylation was promoted by SARS-CoV-2-N protein, resulting in the inhibition of IFN-β signalling in the target cells [38]. When referred to the porcine associated coronavirus, the interaction between IRF3 and TBK1 was impeded by PEDV N protein, leading to the suppression of the type I IFN production mediated by IRF3 activation [60]. To date, fewer studies were investigated about how the host fought against the virus infection and restricted the distinct antagonisms initiated by coronavirus infections. In this study, it has been proved that PDCoV N protein antagonizes IFN-β production by interacting with RIG-I. Upon the translocation of CDC4 from nucleus into cytoplasm post PDCoV infection, the interaction between CDC4 and PDCoV N was confirmed and CDC4 sequestered the interaction between PDCoV N and RIG-I, by which CDC4 plays a positive role in antagonizing the inhibitory effect of PDCoV N on RIG-I-mediated production of IFN. Importantly, a broad-spectrum antiviral activity of CDC4 was confirmed by using the shared mechanisms among the different CoVs from Coronaviridae family, such as TGEV from α-CoV and SARS-CoV-2 from β-CoV.

**Figure 8.**
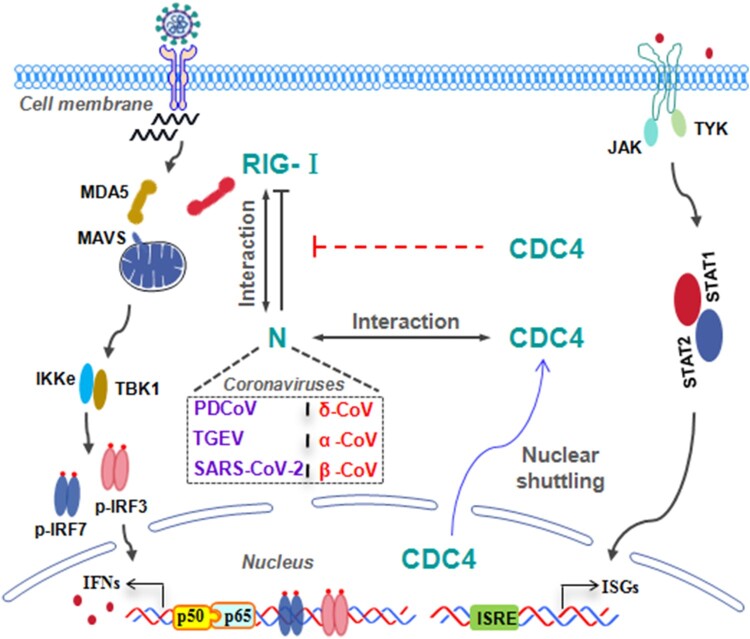
Schematic diagram of broad-spectrum antiviral activity of CDC4 by disrupting CoVs N mediated antagonisms.

In summary, it was well corroborated that CDC4 exerts a broad-spectrum antiviral activity to inhibit the infection of multiple CoVs, including PDCoV, TGEV and SARS-CoV-2. Mechanistically, a novel antiviral strategy of CDC4 was elucidated that CDC4 competes binding with CoVs N proteins to suppress CoVs N mediated antagonism of RLRs associated signalling pathway in the context of diverse coronavirus infections (Figure 8). Our findings offer new insights into the regulation of CoV infections and host antiviral responses.

## Supplementary Material

Supplementary Material.pdf
