# Supplementary material for: Nuclear shuttling of CDC4 mediated broad-spectrum antiviral activity against diverse coronaviruses
Source: Emerg Microbes Infect. 2025 Apr 22;14(1):2493922. doi: 10.1080/22221751.2025.2493922 (PMC12064130; doi:10.1080/22221751.2025.2493922)
Supplement: Supplementary Material.pdf [file TEMI_A_2493922_SM7958.pdf]

Supplementary Material

Nuclear shuttling of CDC4 mediated broad-spectrum antiviral activity against diverse coronaviruses

Mingwei Li [a†], Yang Wu [a†], Jin Tian [a†], Qian Yang [b†], Mingze Gao[a], Yongrui Wang [a], Xuepeng Wang [a], Ju Zhang [a], Yudi Pan [a], Hongyan Shi [a], Da Shi [a], Xin Zhang [a], Jianfei Chen [a*], Longjun Guo [a*], Li Feng [a*]

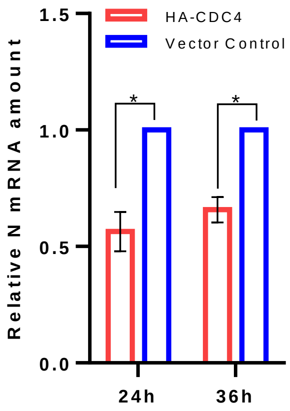

Figure S1. Overexpression of CDC4 inhibits PDCoV replication in ST cells.ST cells were transfected with CDC4 or the vector control for 24 h, and then inoculated with PDCoV (MOI =0.05) for further culture. At 24 h and 36 h post infection, the level of PDCoV replication was evaluated by RT-qPCR. The values are means from three independent infections (means ± SD). *, $P < 0.05$. The P value was calculated using Student' s t-tests.

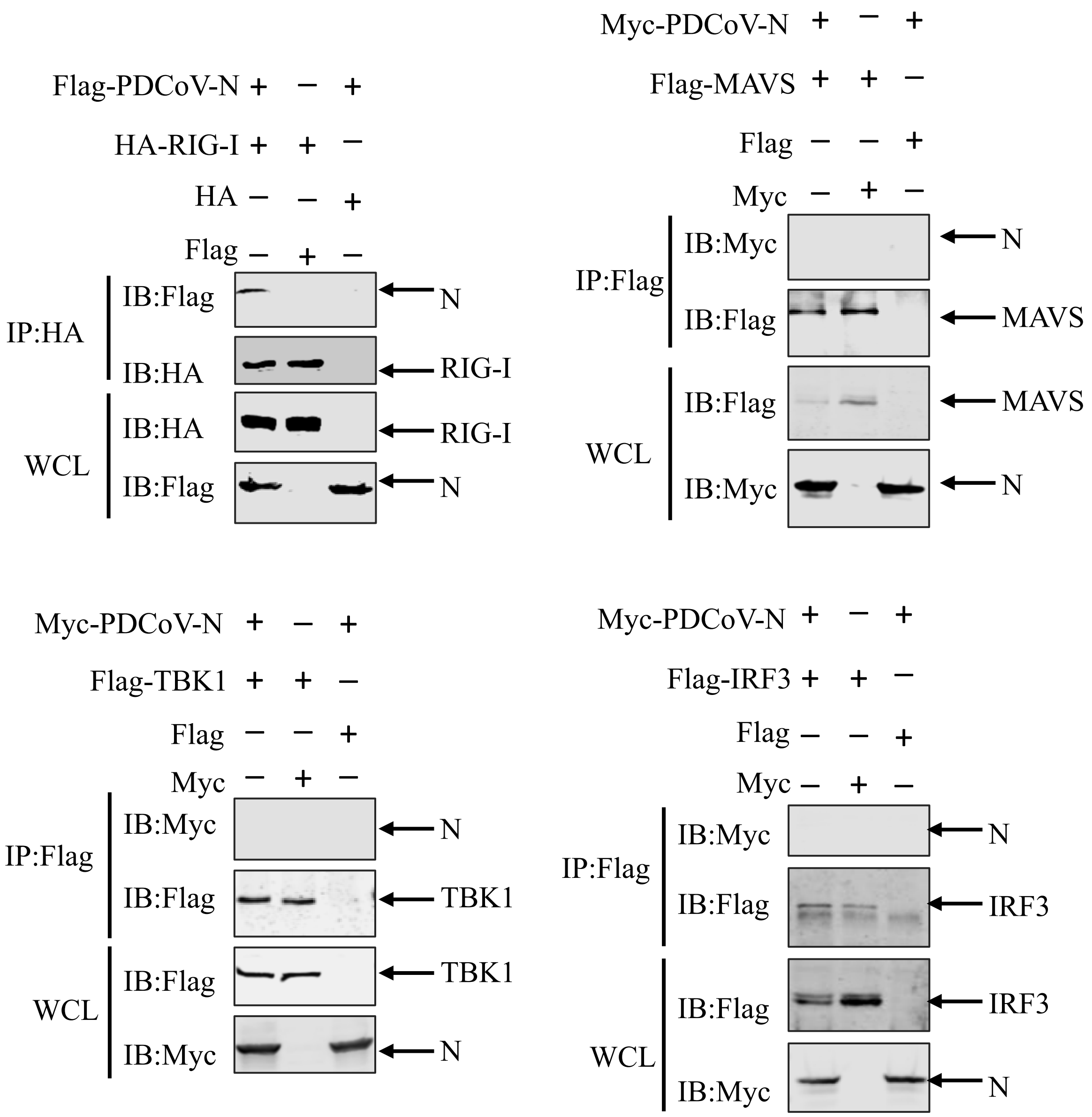

Figure S2. Identification of the interactions between PDCoV N and the key adaptors in the RLRs signaling pathway. HEK 293T cells were co-transfected with PDCoV N and the target plasmids (RIG-I, MAVS, TBK1 and IRF3) for 36 h and cells were collected and lysed for Co-IP analysis with indicated antibodies.

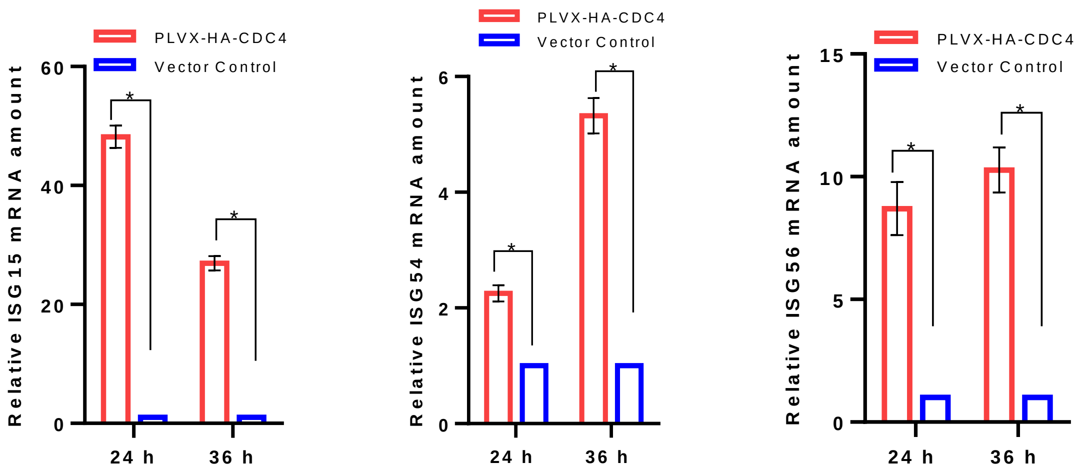

Figure S3. The levels of ISGs were elevated followed by CDC4 in TGEV-infected IPI-2I cells. IPI-2I cells were transduced with CDC4 or the vector control as described above, and inoculated with TGEV for another 24 h. The relative mRNA expressions of ISG15, ISG54 and ISG56 were determined by RT-qPCR. Three independent experiments were performed (means ± SD). *, P < 0.05. The P value was calculated using Student's t-tests.

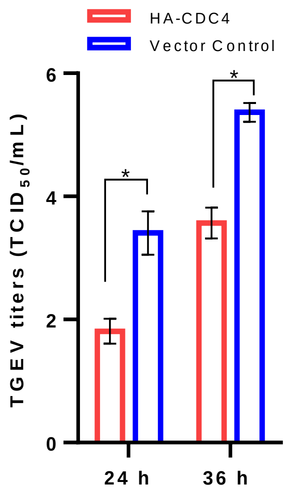

Figure S4. CDC4 suppresses TGEV replication in ST cells. ST cells were transfected with CDC4 or the vector control for 24 h, and then inoculated with TGEV (MOI =0.01) for 24 h or 36 h. The level of TGEV replication was determined by RT-qPCR. The values are means from three independent infections (means ± SD). *, P < 0.05.

Table S1. Primers for PCR used in this study.

Name	Primer sequence (5'→3')
CDC4-F	CCGGAATTCAATCAGGAACTGCTCTCTGTG
CDC4-R	CGGGGTACCTCACTTCATGTCCACATCAAAGT
PLVX-CDC4-F	CCGGAATTCATGGACTACAAAGACGATGACGACAAGAATCAGGAA CTGCTCTCTGTG
PLVX-CDC4-R	CTAGTCTAGATCACTTCATGTCCACATCAAAGTC
ΔF-box-F1	CCGGAATTCAATCAGGAACTGCTCTCTGTG
ΔF-box-R1	AATACCCTCTTCTTTGCATTGAAACTGGGGTGCTAT
ΔF-box-F2	ATAGCACCCCAGTTTCAATGCAAAGAAGAGGGTATT
ΔF-box-R2	CGGGGTACCTCACTTCATGTCCACATCAAAGT
ΔWD40-F	CCGGAATTCAATCAGGAACTGCTCTCTGTG
ΔWD40-R	CGGGGTACCTCATTTCAGCACCTTAGGAGATT

Table S2. siRNA duplexes used in this study.

Name	Sense (5'→3')	Antisense (5'→3')	usage
CDC4 1295#	GCAAAUGUCUGAGGACAUUTT	AAUGUCCUCAGACAUUUGCTT	
CDC4 1157#	GGAGGCGAGGAGAACUCAATT	UUGAGUUCUCCUCGCCUCCTT	IPI-2I cells
CDC4 1096#	GGUUUCAUACACAGUCCAUTT	AUGGACUGUGUAUGAAACCTT	
Negative control (NC)	UUCUCCGAACGUGUCACGUdTdT	ACGUGACACGUUCGGAGAAdTdT	

Table S3. Primers for RT-PCR used in this study.

Name	Primer sequence (5'→3')
qCDC4-F	TGCAAAGTCTCAGAATATACA
qCDC4-R	ATTTCTCTGGTCCACTCCAGC
qPDCoV-N-F	AGCAACCACTCGTGTTACTTG
qPDCoV-N--R	CAACTCTGAAACCTTGAGCTG
qTGEV-N-F	TTTTGTTTGGAAGCTATTGGACT
qTGEV-N--R	CCTTTGGCAAGTGGTATTTGTG
ISG15-F	ATCACCCAGAAGATCGGCG
ISG15-R	TCGAAGGTCAGCCAGAACAG
ISG54-F	CATTGACCCTCTGAGGCAAG
ISG54-R	AGCGTGTCCTATTAGTTCC
ISG56-F	CATACATTTCCACTATGG
ISG56-R	TACTCCAGGGCTTCATTCA
β-actin-F	CTTCCTGGGCATGGAGTCC
β-actin-R	GGCGCGATGATCTTGATCTTC
